# A Rare Complication Developing After Hematopoietic Stem Cell Transplantation: Wernicke’s Encephalopathy

**DOI:** 10.4274/tjh.2014.0412

**Published:** 2015-12-03

**Authors:** Soner Solmaz, Çiğdem Gereklioğlu, Meliha Tan, Şenay Demir, Mahmut Yeral, Aslı Korur, Can Boğa, Hakan Özdoğu

**Affiliations:** 1 Adana Hospital of Başkent University, Department of Hematology, Adana, Turkey; 2 Adana Hospital of Başkent University, Department of Family Medicine, Adana, Turkey; 3 Adana Hospital of Başkent University, Department of Neurology, Adana, Turkey; 4 Adana Hospital of Başkent University, Department of Radiology, Adana, Turkey

**Keywords:** thiamine, Wernicke’s encephalopathy, Hematopoietic stem cell transplantation, Total parenteral nutrition

## Abstract

Thiamine is a water-soluble vitamin. Thiamine deficiency can present as a central nervous system disorder known as Wernicke’s encephalopathy, which classically manifests as confusion, ataxia, and ophthalmoplegia. Wernicke’s encephalopathy has rarely been reported following hematopoietic stem cell transplantation. Herein, we report Wernicke’s encephalopathy in a patient with acute myeloid leukemia who had been receiving prolonged total parenteral nutrition after haploidentical allogeneic hematopoietic stem cell transplantation. To the best of our knowledge, this is the first case reported from Turkey in the literature.

## Abstract (tr)

Tiamin suda çözünen bir vitamindir. Tiamin eksikliği Wernicke ensefalopatisi olarak bilinen, klasik olarak konfüzyon, ataksi ve oftalmopleji ile kendini gösteren bir merkezi sinir sistemi hastalığı olarak karşımıza çıkabilir. Hematopoetik kök hücre nakli sonrasında gelişen Wernicke ensefalopatisi nadiren bildirilmiştir. Bu nedenle haploidentik allojenik kök hücre naklinden sonra uzun süre total parenteral beslenme alan akut myeloid lösemili bir hastada gelişen Wernicke ensefalopatisini sunmak istedik. Bildiğimiz kadarıyla literatürde Türkiye’den bildirilen ilk olgudur.

## INTRODUCTION

Thiamine is a water-soluble vitamin also known as vitamin B1 [1]. Thiamine deficiency can present as a central nervous system (CNS) disorder known as Wernicke’s encephalopathy (WE), which classically manifests as confusion, ataxia, and ophthalmoplegia [1,2]. The disease is most frequently associated with chronic alcoholism, yet it can also occur in relation to other forms of malnutrition or malabsorption such as prolonged total parenteral nutrition (TPN), total gastrectomy, gastrojejunostomy, severe anorexia, or hyperemesis gravidarum [3]. Hematopoietic stem cell transplantation (HSCT) does not seem to have a strong link with WE [4]. To the best of our knowledge, this is the first such case reported from Turkey in the literature and wanted to report this case due to its rarity.

## CASE PRESENTATION

A 19-year-old male patient diagnosed with acute myeloid leukemia was admitted to our hospital for HSCT. After remission had been achieved, he underwent haploidentical HSCT from a sibling donor with a busulfan-fludarabine conditioning regimen. During the conditioning period, the patient was administered TPN, which is routinely used in haploidentical HSCT; however, he developed grade 2-3 nausea and vomiting and could not tolerate TPN. His oral intake was also insufficient, so he received saline solution and glucose-containing intravenous solutions. He gradually recovered from neutropenia on day 13 after HSCT without any adverse events.

He was hospitalized due to diarrhea and vomiting 3 weeks after the transplantation. On follow-up, toxic megacolon and cytomegalovirus positivity were detected, so ganciclovir treatment was started and oral intake was restricted until recovery of intestinal symptoms. Efforts were made to feed the patient by TPN with the aim of meeting his caloric needs although he could not initially tolerate it. He was examined for acute graft-versus-host disease (GVHD); he underwent colonoscopy and pathologic samples were obtained, but this examination did not reveal histological findings of GVHD. Three weeks after his hospitalization, he developed confusion, hallucination, strabismus, and nystagmus. A neurology consultation was therefore done. In his neurologic examination, he was oriented to place and person, but not to time. He had horizontal nystagmus and lateral gaze paralysis in the right eye, his motor power was 4/5, deep tendon reflexes were hypoactive, Babinski reflex was negative bilaterally, he could not cooperate with cerebellar tests, and he could not stand up. Magnetic resonance imaging (MRI) of the brain showed increased signal on T2-weighted and fluid-attenuated inversion recovery (FLAIR) sequences around the aqueductus sylvii and at the medial parts of both thalami (Figures 1a and 1b). A prediagnosis of WE was made based on the patient’s history of inadequate oral intake and TPN use, CNS symptoms, and specific radiologic findings. A blood sample was obtained for testing serum thiamine level to confirm the diagnosis before initiating therapy. Thereafter, 125 mg of thiamine was intravenously administered daily, resulting in a rapid improvement of the CNS symptoms within 48 h of treatment, and parenteral treatment continued for 2 weeks. Serum thiamine level was reported as 7.5 µg/L (normal range: 25-75 µg/L), verifying our diagnosis. During follow-up, his neurologic findings and oral intake gradually improved, and so medical therapy was switched to peroral treatment and maintained with 250 mg of daily peroral thiamine. MRI revealed that the previous increased signal around the aqueductus sylvii and at the medial parts of both thalami on T2-weighted and FLAIR sequences had significantly diminished (Figures 2a and 2b). Informed consent was obtained.

## DISCUSSION AND REVIEW OF THE LITERATURE

Neurological complications are fairly common in patients undergoing HSCT and are present in 30%-39% of cases [5]. These complications may be of infectious, cerebrovascular, toxic, immune-mediated, or metabolic origin [5]. Additionally, several drugs routinely used during HSCT are associated with neurological abnormalities, including cyclosporine A [5] and tacrolimus [6]. Used alone or in combination with other agents, methylprednisolone and ganciclovir may be responsible for neurological findings, including disorientation, altered mental status, visual disturbance, and coma [5]. We think that we saved some time in making a differential diagnosis by examining serum tacrolimus level to exclude drug toxicity and cerebrovascular causes.

WE is characterized by the triad of altered mental status, ataxia, and ophthalmoplegia, but only 16% of patients present with the full classic triad of symptoms [5]. Mental status changes are the most frequent findings in these patients (82%), followed by ocular findings (29%) and ataxia (23%) [5]. Ocular signs, including ophthalmoplegia, horizontal and vertical nystagmus, and conjugate gaze palsies, are the hallmark of WE [3]. Although almost all WE patients show some degree of improvement after initiation of thiamine replacement, only about 20% recover completely [4]. Furthermore, mortality increases dramatically when treatment is delayed [4]. According to the guidelines of the European Federation of Neurological Societies, total thiamine in blood samples should be measured immediately before thiamine administration to confirm suspected or manifest WE and MRI should be used to support diagnosis [7]. Fortunately, we could make a timely diagnosis based on clinical and radiological findings and supported by decreased thiamine level thereafter, and thus we could prevent mortality.

Patients receiving long-term TPN and glucose-containing intravenous solutions require larger amounts of thiamine to metabolize their carbohydrate intake, which can rapidly deplete thiamine stores [3]. Studies show that a state of depletion could develop within 18-20 days in patients receiving a strict thiamine-free diet [5]. Almost all published reports, except for one, concluded that prolonged TPN was the primary risk factor for HSCT-associated WE [4]. Our patient had received TPN for approximately 4-5 weeks in total. TPN includes multivitamin and mineral supplementation in our routine treatment protocol. However, we could not administer it in this patient due to temporary lack of the concerned drugs in the pharmacy of the hospital. The only other suggested cause was the use of busulfan in the conditioning regimen [4]. Similarly to data in the literature, our patient received busulfan in the conditioning regimen and thiamine-free TPN, and symptoms of WE emerged from day +45.

Many authors have recommended the use of a thiamine supplement for prophylaxis against WE [4]. However, an earlier publication from a Brazilian group reported 8 patients who died after developing WE despite receiving prophylactic thiamine (50 mg/day) [4]. Further studies are required to decide on an effective prophylactic dose of thiamine and to determine whether thiamine prophylaxis is effective in the prevention of WE in HSCT patients [4]. This case taught us the vital importance of vitamin supplementation in patients who need long-term TPN. Based on these findings, we reviewed our institutional policy about vitamin supplementation in TPN and began adding water-soluble vitamins into TPN solutions individually if combination preparations were not available in the pharmacy of the hospital.

There are not routine recommendations for initial CNS evaluation and management of the rarely occurring WE [4]. However, WE is a neurological emergency [8]. Therefore, WE should be considered in HSCT patients, because cancer patients are at high risk of this acute encephalopathy due to chronic malnutrition, chemotherapy-induced nausea and vomiting, and consumption of thiamine by rapidly growing tumors [8].

In conclusion, differential diagnosis should consider WE for patients who undergo HSCT and develop neurological symptoms. Early treatment prevents high morbidity and mortality. Therefore, thiamine supplements should be administered to patients at high risk for WE.

## Figures and Tables

**Figure 1a:**
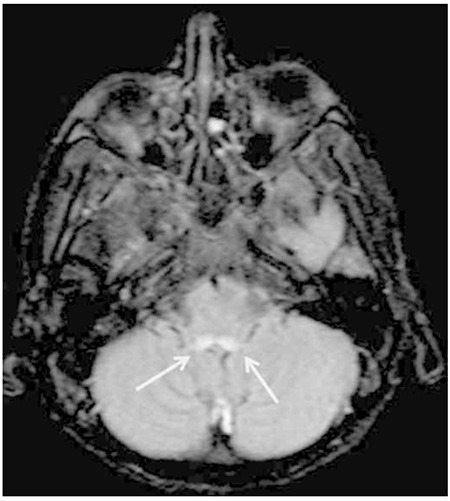
Axial fluid-attenuated inversion recovery magnetic resonance imaging images of the brain demonstrating the increased signal around the aqueductus sylvii.

**Figure 1b:**
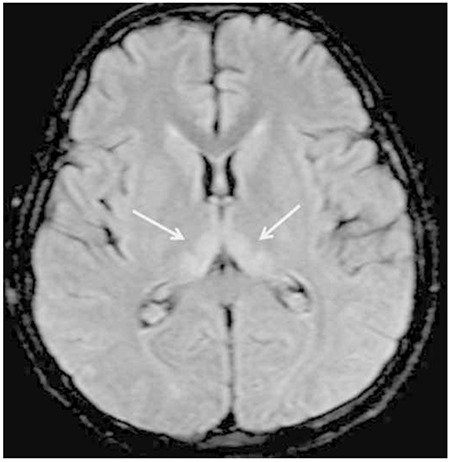
Axial fluid-attenuated inversion recovery magnetic resonance imaging images of the brain demonstrating the increased signal at the medial parts of both thalami.

**Figure 2a:**
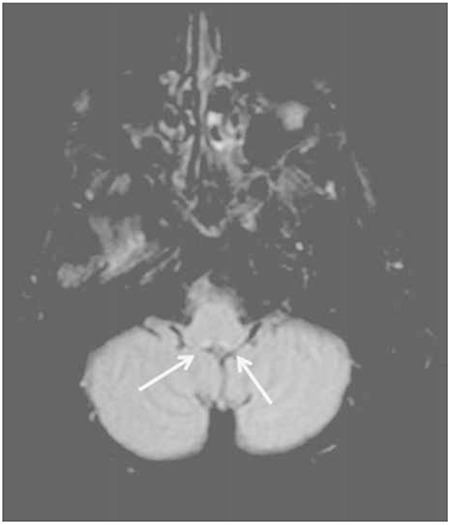
Control magnetic resonance imaging 2 weeks after the onset of the symptoms; fluid-attenuated inversion recovery image showing the diminution of increased signal around the aqueductus sylvii.

**Figure 2b:**
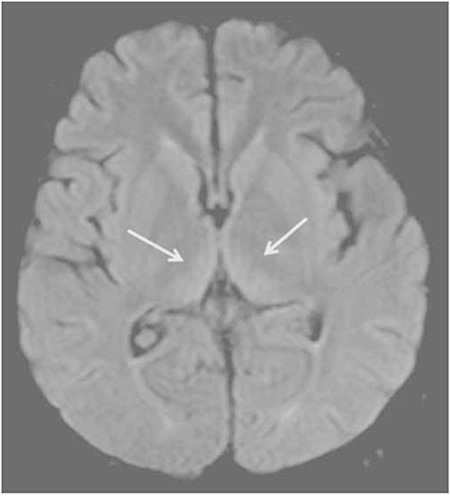
Control magnetic resonance imaging 2 weeks after the onset of the symptoms; fluid-attenuated inversion recovery image showing the diminution of increased signal at the medial parts of both thalami.
